# Addressing the “It Is Just Placebo” Pitfall in CAM: Methodology of a Project to Develop Patient-Reported Measures of Nonspecific Factors in Healing

**DOI:** 10.1155/2013/613797

**Published:** 2013-12-19

**Authors:** Carol M. Greco, Ronald M. Glick, Natalia E. Morone, Michael J. Schneider

**Affiliations:** ^1^Department of Psychiatry, University of Pittsburgh School of Medicine, UPMC Center for Integrative Medicine, 580 S. Aiken Avenue, Suite 310, Pittsburgh, PA 15232, USA; ^2^Department of Psychiatry and Physical Medicine and Rehabilitation, University of Pittsburgh School of Medicine, Medical Director, UPMC Center for Integrative Medicine, 580 S. Aiken Avenue, Suite 310, Pittsburgh, PA 15232, USA; ^3^Department of Medicine, Division of General Internal Medicine, Center for Research on Health Care, University of Pittsburgh School of Medicine, 230 McKee Place, Suite 600, Pittsburgh, PA 15213, USA; ^4^Department of Physical Therapy, University of Pittsburgh School of Health and Rehabilitation Sciences, 6035 Forbes Tower, University of Pittsburgh, Pittsburgh, PA 15241, USA

## Abstract

CAM therapies are often dismissed as “no better than placebo;” however, this belief may be overcome through careful analysis of nonspecific factors in healing. To improve trial methodology, we propose that CAM (and conventional) RCTs should evaluate and adjust for the effects of intrapersonal, interpersonal, and environmental factors on outcomes. However, measurement of these is challenging, and there are no brief, precise instruments that are suitable for widespread use in trials and clinical settings. This paper describes the methodology of a project to develop a set of patient-reported instruments that will quantify the nonspecific or “placebo” effects that are in fact specific and active ingredients in healing. The project uses the rigorous instrument-development methodology of the NIH-PROMIS initiative. The methods include (1) integration of patients' and clinicians' opinions with existing literature; (2) development of relevant items; (3) calibration of items on large samples; (4) classical test theory and modern psychometric methods to select the most useful items; (5) development of computerized adaptive tests (CATs) that maximize information while minimizing patient burden; and (6) initial validation studies. The instruments will have the potential to revolutionize clinical trials in both CAM and conventional medicine through quantifying contextual factors that contribute to healing.

## 1. Background

Complementary and Alternative Medicine (CAM) studies that fit the current “gold standard” of randomized, double-blind, placebo, or sham controlled designs frequently do not show an effect of the active treatment above that of the supposedly “inert” placebo [1–3]. This is often pointed to as evidence that CAM approaches are not active treatments; they show benefit only or largely through the placebo effect. Perhaps such “negative results” are due to the limited ability of placebo controlled research designs to capture important aspects of healing as they relate to health outcomes. The negative results may not be a placebo response; rather, they may reflect nonspecific factors that contribute to a positive response in both active and placebo interventions. It is important to understand these nonspecific contributors, as they may overlie the active, unique benefits of a particular CAM therapy, leading to the erroneous conclusion that the treatment is ineffective.

In its third strategic plan, published in 2011, the National Center for Complementary and Alternative Medicine (NCCAM) took stock of its first 13 years of operations and set goals for future research directions [4]. This document spoke for the field, reflecting and responding to shortcomings in our current research designs and suggesting directions the field must move toward for greatest relevance and impact. The NCCAM document focused on some of the challenges of trying to fit the study of complementary medicine modalities into the “gold standard” RCT model that was developed more specifically for drug and biomedically-oriented treatment studies. Such challenges include the historical focus of RCTs on standard clinical outcomes as opposed to patient centered outcomes, and the focus on “efficacy over placebo” rather than real world effectiveness. As an example, they cite a series of large acupuncture clinical trials in Germany that compared acupuncture, sham, and usual care, finding statistically significant and clinically meaningful differences between both needle groups and usual care but only a marginal difference between the 2 acupuncture groups. This raised the question of specific versus nonspecific effects of treatment. Rather than dismissing this as “all placebo,” they note that

*Better understanding of the contributions of both specific and nonspecific effects influencing outcomes and the potential for insight into exploitation of either or both to improve symptom management or general health and well-being is needed.*

The difficulty of clarifying specific and nonspecific effects was raised by Yeh and colleagues in a commentary on a clinical trial of Tai Chi for fibromyalgia [5]. “The authors dutifully suggest that a sham Tai Chi intervention would have been desirable as a control. Ideally, a placebo control matches all aspects of the therapeutic intervention except for the “active” element of that intervention. But what is the active element of a complex, multicomponent therapy such as tai chi? Is it rhythmic exercise, deliberate and deep breathing, contemplative concentration, group support, relaxing imagery, a charismatic teacher, or some synergistic combination of these elements? If so, would the matched control include awkward movements, halted breathing, participant isolation, unpleasant imagery, or a tepid teacher? Would the resulting sham intervention be credible, valid, or even genuinely inactive?” The authors point out the complexity of CAM treatments such as Tai Chi and the sheer impossibility of providing an inert placebo as a control.

CAM researchers and practitioners often embrace a holistic approach that recognizes the importance of many factors contributing to healing. Healing is a complex process involving “physical, mental, social, and spiritual processes of recovery, repair, renewal and transformation that increase wholeness, and often (though not invariably), order and coherence” [6]. Healing may or may not involve “cure” or the resolution of a diagnosis or disease. Although there is growing recognition of the importance of the within-person, social, and environmental contexts involved in health, such influential factors are seldom measured, and there have been few attempts to assess patients' perceptions of healing [7–9].

Whether or not conventional medicine clinicians and researchers acknowledge the importance of the healing context, their patients are nevertheless influenced by their own attitudes, outcome expectancies, and perceptions of their provider. Recent mechanistic studies of the placebo effect and placebo response indicate that the endogenous opioid, serotonin, and dopaminergic systems are involved [10]. The placebo effect stems from highly active processes in the brain that are mediated by psychological mechanisms such as expectation and conditioning [11]. Rather than being unknowable and something to minimize, “placebo” and factors that are not specific to the treatment are important to measure, both in CAM and conventional medicine trials.

There are several reasons why measuring nonspecific factors is important. First, for CAM and conventional medicine researchers, nonspecific factors can be considered as moderating factors that influence treatment outcomes. Quantification of such moderators will allow researchers to statistically adjust for them, leading to more accurate assessment of the effects of active treatments and comparison interventions. This is analogous to adjusting for moderating factors such as age in epidemiologic studies. Additionally, precise measurement of a variety of nonspecific factors can advance knowledge regarding which factors are important for which treatments and types of patients. For example, the patient's perceptions of their relationship with the provider may be more important for those with a chronic, complex condition rather than an acute illness. The insight gained will allow clinicians and researchers to identify and target the important factors that should be enhanced in order to improve health outcomes, both in research settings and in clinical care settings.

In this paper, we present the methodology of an ongoing instrument development project that is creating brief, precise, patient-reported measures of nonspecific factors that influence healing. A set of patient-reported measures to quantify the nonspecific factors in healing will advance CAM research, and may ultimately improve research trial design in general. There are several methodological challenges inherent in measuring contextual factors of healing, however. Challenging questions such as what areas to measure, how best to measure them, and how to maximize information while minimizing patient burden are important to address. We describe the challenging questions, and explain how the projects' methodology is uniquely suited to address the challenges.

## 2. Methodological Challenge 1: What to Measure?

An initial challenge in the measurement of healing contexts is figuring out what to measure. Depending on the research setting and design, the type of treatment, and the amount of contact between patient and provider, a variety of factors may be important to measure. The areas reported in the literature as being important to healing can be broadly categorized into two domains: *Patient Attitudes*, and *Patient-Provider Relationships*. Patient Attitudes include both “intrapersonal” or “patient” characteristics, and attitudes regarding current treatment, CAM, and conventional medicine. Patient-Provider relationship includes the patients' perception of connection with their treatment provider as well as their perception of the overall healing environment.

Within CAM, certain attitudes and characteristics of the patient have been found to predict outcomes and may therefore be important to measure in trials. For example, patient self-report of spirituality predicts outcomes of CAM [12] as well as the likelihood of CAM use [13]. Similarly, attitudes of optimistic hope and desire for improvement are associated with larger effects of placebo treatments in CAM and conventional medicine trials [14, 15]. Taking an active role in caring for one's self may also contribute to healing [9]. Concordance between treatment and patients' beliefs about the treatment was found to influence the outcomes in a study of flower essences [16]. The patient characteristic of openness to CAM was found to promote CAM treatment seeking [17] as well as predict benefit from CAM therapy [12].

Patients' expectations regarding their treatment are often assessed in nonpharmacologic trials. Historically, the purpose of such measurement is to document that the treatment and control conditions are equally credible. More recently, however, participants' expectations of benefit have been linked to treatment outcomes [9, 15, 18, 19]. In several studies, experimental manipulation of expectancies has led to pain reduction and changes in physiologic processes such as heart rate and blood pressure [20, 21]. In a study of massage, acupuncture, and self-education in 262 patients with chronic low back pain (CLBP), those who had the largest expectation of change for a specific treatment had the largest therapeutic benefit, with an odds ratio of 5.3 for improvement when other factors were controlled for [22]. A recent open-label study of placebo without deception, for irritable bowel syndrome, lends further support to the role of positive expectations in outcomes [23]. Subjects who received clearly labeled Placebo tablets were informed that despite having no active ingredients, placebos have been shown to induce mind-body effects. Of the open-label placebo patients, 59% reported adequate symptom relief, whereas 35% of the control group, who received attention from their healthcare provider, reported symptom relief to be adequate. The authors concluded that expectation of a positive outcome contributed to the significant clinical outcome and at least partially explained the placebo response.

Another widely recognized factor that may contribute to healing is the patient-provider relationship. The mental health literature supports the importance of perceived therapist expertise, shared mission or goals, and perceived bond or connection between patient and provider for enhancing outcomes [24]. A CAM study designed to dismantle patient-provider relationship components of the placebo effect used three conditions: wait-list, sham acupuncture with limited interaction with the provider, and sham acupuncture enhanced by provider warmth. The study showed a dose-response relationship, with adequate symptom relief of irritable bowel symptoms at three weeks in 28% of waiting list, 44% of limited interaction sham acupuncture, and 62% of sham enhanced by provider warmth [18]. However, not every patient is equally responsive to provider warmth. In the previously mentioned study, only those scoring high on an extraversion scale showed greater symptom improvement with warm providers [25]. While clinicians, researchers, and hospital systems are growing to recognize the importance of the patient-provider relationship, quantifying this important area is complex: the patient-provider relationship can be assessed by clinicians, patients, or through observer rating scales. A single set of questions that can be applied to any setting and treatment is needed.

In addition to patient attitudes and expectations, and interpersonal factors, environmental factors may be important to assess as potential contributors to healing outcomes [26–29]. These may include rituals of engaging in treatment, and entering into a “healing environment” that has a pleasant physical setting and caring social atmosphere. Although these factors are typically assessed in patient satisfaction questionnaires, they are rarely included as moderators of treatment outcome. Measuring environmental effects can also be challenging because the patient may not be aware that the provider has taken care to provide a soothing environment by careful choice of color, natural light, live plants, and so forth. Yet it is important to capture the effect of environment on the patient because of its potential influence on treatment outcome.

## 3. Methodological Challenge 2: How to Measure Nonspecific or Contextual Factors in Healing?

There are several considerations that go into the assessment of the impact of contextual factors in healing. As noted, there are a number of domains that may contribute to healing, independent of the specific treatment. Some domains may be more salient in certain interventions. For example, one would assume that perceptions of the patient-provider relationship would be more important in a mind-body encounter than in a chiropractic manipulation treatment. While it might be tempting to tailor a measurement instrument to the treatment modality, this would leave us in a position in which one cannot easily compare the assessments from one study to another. Consequently, it is preferable to have one set of instruments that can be employed and validated across different kinds of CAM trials.

Many instruments assessing factors of interest are already in existence. However, it is difficult to determine which to use when designing a study. Among the domains mentioned above, there is variability in the quantity and quality of measures available. While some domains like optimism/positive attitudes have extensively validated measures such as the Life Orientation Test-Revised (LOT-R) [30], other domains such as the physical environment or attitudes towards CAM have few or no validated measures. This variability in quality leaves researchers with questionnaires that can be lengthy or repetitive, or that do not adequately measure the constructs of interest. Questionnaires may have great variability in language, clarity, and response categories, which may weaken validity as well as comparability of results across studies. Important considerations include how was the instrument designed and validated? Was it developed via expert opinion? Were patients' views included? Was it validated on a small sample of patients? Was it tested only on a limited population such as University students?

A good example of the “how to measure” challenge in assessing contextual factors in healing is the domain of treatment expectations. Some RCTs of CAM therapies have shown that treatment expectations are highly correlated with treatment outcome, yet other studies have not shown this same association. This may be due to real differences in the importance of expectations across treatments and across patient populations. However, there are several possible methodological explanations for this inconsistency as well. The first is simply the lack of a standardized measurement instrument for treatment expectations, which leads to different trials using different expectation instruments. Secondly, researchers may decide to develop their own questions and rating systems for “measuring” treatment expectancy. In both of these cases, this leads to a lack of consistency across trials with respect to the measurement of treatment expectation. Lastly, there is a lack of consistency across trials for the time point(s) at which treatment expectation is measured within those trials. Some trials measure treatment expectation only at baseline, whereas other trials measure expectation at multiple time points during and after the active treatment period. It is likely that the association between treatment expectation and outcomes varies depending on the time point at which it is measured, complicating the comparisons across studies. The issue of time points, or when to measure, is an important but often neglected part of the “how to measure” challenge.

Unfortunately, researchers often take an existing instrument and change the wording of the items to presumably fit their research trial. This approach may completely skew the psychometric properties of the original instrument and lead to poor measurement characteristics of the modified questionnaire. For example, the credibility/expectancy questionnaire (CEQ), questionnaire published by Borkovec and Nau [31], was originally designed to measure undergraduate psychology students' expectations about different psychotherapeutic approaches to reduce test performance anxiety. Over the years, we have seen the wording of items in this questionnaire completely altered for use in trials that are studying many interventions for many conditions, from behavioral treatments for stress to somatic treatments such as acupuncture for back pain. Only recently, one such modification of the CEQ has been validated for use in back pain trials [32, 33].

Although there is strong support for the role of expectations in outcome, there is no single instrument that measures treatment expectations in a general way. The use of treatment-specific instruments and poorly validated adaptations of existing questionnaires make it difficult to compare the role of patient expectations across studies. A single brief measure that is applicable to a broad range of treatments and conditions would standardize the measurement of expectation and remove this barrier. To best meet the needs of future researchers and their patients, instruments designed to measure or quantify nonspecific factors should be general, brief, and well validated.

## 4. Methodological Challenge 3: How to Maximize Information without Excessive Burden on Participants?

A general barrier that is faced by all investigators is balancing the measurement of important outcomes and moderating and mediating factors with the need to keep subject burden to a minimum. Subject burden is a major concern in research studies and lengthy questionnaires are potentially onerous. Adding more questionnaires in the hopes of capturing unknown important factors may result in disgruntled research subjects or even accelerated drop-out rates. Too many questions may be a threat to validity; it is easy to imagine that research subjects' responses to questions 200–250 are less carefully considered than their responses to questions 1–30. To best meet the needs of future researchers and their patients, instruments designed to measure or quantify nonspecific factors should be both widely applicable, simple for patients to complete, and brief.

## 5. Meeting the Challenges: Methodology of the Healing Encounters and Attitudes Lists (HEAL) Instrument Development and Validation Study

The challenges of measuring nonspecific or contextual factors are significant but not insurmountable. We report on the methodology of a project designed to meet these challenges and bring a set of useful and precise tools to CAM and conventional medicine researchers and clinicians. The overall objective of this project, entitled *The Healing Context in CAM: Instrument Development and Initial Validation *(NCT01266304, NCT01904838), is to develop and test a set of efficient patient-report instruments to measure CAM-relevant contextual factors that are important in healing. The healing context, which includes such factors as patients' beliefs and expectancies and their perceptions of the patient-provider relationship and the treatment environment, may account for much of what is known as the “placebo response.” A set of self-report tools to assess contextual factors that contribute to healing has the potential to revolutionize efficacy trials of CAM and also conventional medicine.

This project uses the rigorous instrument development methodology of the NIH Roadmap Initiative, Patient Reported Outcomes Measurement Information System (PROMIS) (http://www.nihpromis.org/). PROMIS uses modern psychometric methods and classical test theory to develop highly reliable, precise, and flexible and efficient assessment tools to measure patient-reported health status. To date, the PROMIS initiative has developed 34 instruments for adults and 6 for pediatric patients, in areas ranging from Emotional Support to Global Health. Notably, the PROMIS methodology results in instruments that are unique in that they are standardized to be comparable across diseases, they can be used across treatments, and they are reliable and valid for patients across a wide range of age, literacy level, and level of physical function. Thus, the methods are ideally suited for developing tools for widespread use in measuring nonspecific factors of healing.

## 6. Meeting Methodological Challenge 1: What to Measure

In order to fully explore all of the potential important contextual factors in healing, a broad approach is necessary initially. The comprehensive approach used by our team was based upon PROMIS methodology, and included the voices of expert clinicians and patients, as well as a careful review of the scientific literature. We conducted semistructured interviews with 22 CAM clinicians and clinician-researchers regarding their opinions about important nonspecific factors in healing. We also consulted an expert on optimal healing environments, Dr. Wayne Jonas, director of the Samueli Institute. The interviewees noted the importance of certain patient characteristics, such as positive outlook, spirituality, self-efficacy for caring for their own health, beliefs about CAM, and treatment preferences and expectations. Important patient-provider interaction factors included the therapeutic partnership, respect for patients' cultural background and beliefs, the conveyance of hope, and empowerment. Also noted were the overall importance of the treatment team and the environment, and the perceived rituals of the healing encounter.

We conducted 6 focus groups, with 6–9 racially and educationally diverse patients in each, in the Pittsburgh, PA, area: 2 groups of patients recruited from an integrative medicine clinic, 2 focus groups of patients from a conventional medicine clinic, and 2 focus groups of community members who had participated in CAM and/or conventional medicine treatments. The focus group scripts invited participants to share their opinions on, for example, what characteristics are most helpful in a health care provider, and how can patients themselves influence their own healing. Similar themes were identified by CAM, conventional, and community patients. Provider characteristics of empathy, openness, confidence, and respect for patients were seen as contributing positively to healing. The focus group patients acknowledged that their own optimism, patience, and ability to take an active role in their health were important attitudes and characteristics that contributed to their healing. They noted key features of a healing environment, such as a friendly, welcoming staff, cleanliness, and a restful and professional atmosphere. They also noted the importance of teamwork, in which the provider may be the “team captain” but the patient is also responsible for follow-through with treatment, communicating with the provider, and taking care of themselves.

**Figure 1:**
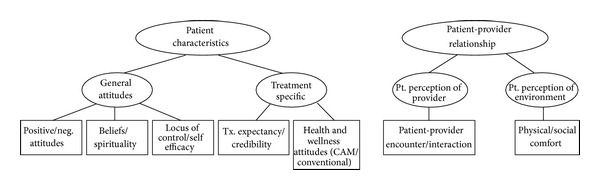
Model of domains important in healing.

From the expert interviews and patient focus groups, sets of key words and key concepts were developed. Based upon the concepts expressed by clinicians and patients, and from the literature on placebo and nonspecific factors that influence health outcomes, we developed an initial conceptual model of domains important to healing (Figure 1).

Once the initial model was formulated, the next step in developing our set of HEAL measures was to fully explore any relevant publications in each of the model's domains. The purpose of this was 2-fold: (1) to capture any relevant conceptual material that might further inform the model and (2) to find and evaluate any existing questionnaires relevant to the domains in the model. This effort began with a comprehensive search for any existing instruments that measure the same or similar domains, and evaluate the individual items. Using the model as a guide, our research librarian coinvestigator developed search strategies based upon relevant keywords, integrating the opinion of our CAM coinvestigators as well as instrument development experts' opinions. The search strategies were applied to 9 scientific databases for each of the domains in the model, yielding a total of over 12,000 abstracts. Our project's team, consisting of CAM researchers and instrument development experts, then reviewed every abstract and flagged those that had potentially useful conceptual content or psychometric information relating to existing instruments. The number of abstracts ranged from 479 for the domain of the Health Care Environment to 3865 for the domain of locus of control/self-efficacy. As we reviewed the abstracts, we were able to identify and obtain any full length articles that contained existing questionnaires, or articles whose content could be used to further develop the conceptual model. Because an iterative approach was used that considered experts opinions, patients' opinions, and careful integration of any other concepts in the scientific literature, we were reasonably clear that our working model incorporated the important nonspecific or conceptual factors of healing.

## 7. Meeting Methodological Challenge 2: How to Measure Nonspecific Factors in Healing 

The overall goal of the HEAL project is to quantify the nonspecific contributors to healing from the patient's perspective, and to do this through precise, efficient, easy-to-use questionnaires. Many questionnaires on the topics of the patient-provider relationship, locus of control, treatment expectations, and the other model domains already exist. In fact, through the literature searches and abstract reviews we identified a total of 535 unique instruments related to the domains of our model. However, many of the instruments are actually disease-specific, treatment-specific, are very lengthy, or are physician or observer ratings rather than patient reports.

The PROMIS methodology for instrument development includes developing “banks” of items from which final questionnaires may be created. As an initial step in creating our HEAL item banks, all of the items from the 535 existing instruments were entered into a database or initial item “pool” which was comprised of over 17,000 items. Our team of CAM investigators and instrument development experts formed subgroups of 2–5 persons per domain to define conceptual meaningful categories within each domain of the model, and sort the existing items into the categories. This was an iterative process, meaning that the categories themselves could be further refined based on information gained during review of the specific items. As an example, the domain of Healthcare Environment Perceptions included factors of Process of Care and Physical Space. Within Physical Space, items could be sorted into “bins” such as general comfort of the waiting room, privacy of the treatment room, and sensory elements such as quietness, and so forth. The teams of investigators sorted the items, and during this process they also engaged in eliminating any items that were redundant with other items, were vague, or were too narrow in focus. This phase of the project also included rewriting any items that were difficult to understand, or that contained more than one concept (e.g., “double-barreled” items).  Table 1 provides examples of problematic items and our efforts at rewriting to increase clarity and simplicity. Our intent was to end up with “item banks” for each domain that included a range of questions for each of the conceptual areas. During this editing and rewriting phase, items were carefully worded so that the same sets of response categories could be used. We used the following 2 types of response categories: frequency (five responses ranging from “never” to “almost always”) or intensity (five responses ranging from “not at all” to “very much”). Overall, the aim was to keep both the question and the response set very simple and easy to use.

It is crucial that patient-reported assessment tools be clear and understandable to patients. Therefore, once the item banks were revised, each item was reviewed by patients for clarity. We conducted individual “cognitive interviews” with 42 CAM and conventional medicine patients of diverse races and ethnicities, and diverse educational backgrounds. In a cognitive interview, the patient is asked to think aloud while viewing and answering items, one-by-one. As the patient speaks, a research associate takes precise notes, and queries the patient not only on the item but also on the appropriateness of the response category. Each item was reviewed by at least 6 patients, at least one of whom had a low level of education (high school or less). This process revealed several items that were difficult for some respondents to understand as they were intended, or remained confusing. These items were then reviewed by the principal investigator and research staff, and were rewritten or eliminated. Any rewritten items were reviewed again by patients in further cognitive interviews. Thus, the cognitive interview stage resulted in refinement and reduction of the item banks. After the methodical and iterative process of revising and refining the seven item banks, a total of 296 items were retained. The item banks for the individual domains ranged in size from 34 items assessing Health and Wellness attitudes (CAM and conventional medicine) to 61 items assessing the Patient-Provider Encounter.

An additional set of steps toward finalizing our HEAL tools involved the use of statistical calibration methods. Not only must items be clear and understandable to patients but also they must be informative about the HEAL domain areas. Consistent with the methodology of PROMIS [34], Classical test theory (CTT) and modern psychometric methods of item response theory (IRT) and tests of Differential Item Functioning (DIF) are used to determine which items should be retained from a statistical and measurement standpoint. In order to conduct these analyses, we tested the sets of items on an internet sample of 1400 adults who had experienced CAM or conventional treatments currently or within the past year, and on 127 patients at the Center for Integrative Medicine and 131 conventional medicine patients at University of Pittsburgh Medical Center (UPMC). The internet sample was representative of the US population in terms of age and race and was collected through YouGov (http:/www.yougov.com/), an internet polling company.

Determining which items in our HEAL banks to retain from a quantitative or statistical standpoint is an iterative process, as were the qualitative methods used in the development of the item banks. This phase of the HEAL instrument development project is currently underway. The data from the calibration sample will be subjected to CTT methods of exploratory factor analysis (EFA) and confirmatory factor analysis (CFA) to determine factor loadings of items onto our model's domains. Based on results of CTT analyses, items may be dropped due to failure to load on any domain or construct. Additionally, at this stage, the item factor loadings may inform our original model, leading to possible model revision. One goal of this analytic step is to determine unidimensionality of each domain. Next, IRT methods are applied to determine whether items provide sufficient information about the underlying domain or construct. At this stage, items may be dropped if they that are not informative about the subject's level on the construct of interest. In the case of a HEAL assessment tool, an item may be dropped if it does not provide any information about, for example, the subject's perception of their encounter with their healthcare provider. In the case of educational tests such as the Scholastic Aptitude Test (SAT), only items that provide information about ability are used. To further determine the best items for use in our HEAL tools, tests of DIF are applied. The purpose of DIF analyses are to confirm that items perform consistently (i.e., do not perform differently) across individuals based on parameters such as age, gender, or education level. These series of analyses will result in retaining only the items that best fit the conceptual areas, and that do not perform differentially based upon demographic features. Thus, the quantitative analyses will result in further refinement and reduction in the item banks.

During the calibration phase of our set of HEAL tools, the local clinical samples also completed “legacy” or well known and frequently used instruments assessing perceptions of their provider, optimism, and other areas within our model, as well as a clinical global impression [35] of improvement question collected several weeks after initiating treatment. The local samples include patients who were currently participating in CAM and conventional treatments for a variety of medical and mental health issues. The data on perceived treatment success will provide clues as to the predictive validity of our HEAL tools once they are finalized. The data on legacy instruments will allow our team to estimate concurrent validity of our HEAL tools. Legacy instruments are expected to exhibit moderate to high correlations with the corresponding final HEAL domain instrument. A future stage in evaluating the validity of the finalized HEAL set of instruments will include evaluating them in a sample of 200 patients participating in CAM and conventional treatments for chronic back pain in 2014-2015, but the calibration sample data provides useful initial validity information in the meantime.

The methods used in the creation of the HEAL tools meet modern standards for instrument development and will result in questionnaires that are easy for patients to complete and very informative about the underlying constructs or domains that are presumed to be within-person and contextual factors of healing. Once finalized, the HEAL tools will be in the public domain and will be available to researchers and clinicians.

## 8. Meeting Methodological Challenge 3: How to Maximize Information While Minimizing Burden on Participants

The balance between gathering the most information possible without burdening patients excessively is a challenge that all clinical trial researchers face. Inserting the assessment of nonspecific, contextual factors represents an added complication to the typical issues of primary and secondary outcome measure assessment. Once the HEAL set of assessment tools are finalized, will they be feasible for widespread use?

The HEAL assessment tools, like the PROMIS instruments, will be concise yet will provide precise information. The IRT-calibrated sets of items will be used to produce computerized adaptive tests (CATs). In CAT, the presentation of items is tailored individually to respondents and their levels of the underlying construct (e.g., patient-provider bond). Based on a respondent's answer to the first question, the most informative follow-up question will be presented next. In other words, the assessment tool “adapts” to the patient as questions are answered. Because the CAT software uses the IRT calibrations, relatively few items will need to be answered in order to fully determine the respondent's standing or “level” on the construct [36]. Therefore, even though there may be as many as 30 or more potentially useful items in the domain's item bank, usually the respondent will only need to answer 3–6 items. Furthermore, the iterative process of writing, rewriting, and revision based on cognitive interviews with diverse patients results in items that are very simple, straightforward, and easy to answer. Typically, at least 6 items can be answered per minute. Therefore, adding a set of HEAL tools is not expected to add undue burden on patients.

In addition to CATs, the IRT-calibrated sets of item can be used to produce Short Forms. Short Forms are fixed (not adapting) sets of 4–10 items that provide information consistent with CATs. Reise and Henson [37] showed that a fixed Short Form, which consists of items most often administered at the start of CAT, performs equally well compared to the CAT version of the same length. Fixed Short Forms based on CAT simulations optimize total test information for most individuals. When the HEAL project's IRT calibration phase is complete, we will develop Short Forms in addition to CATs. The advantage of Short Forms is that if needed, they can be administered on paper or through web-based platforms without the CAT software, whereas the HEAL CATs, when finalized, will need to be administered through the Assessment Center (https://www.assessmentcenter.net/). Assessment Center is an online data collection tool, currently free, through which the PROMIS CATs are administered. Whether administered as CATs or Short Forms, the entire set of HEAL assessment tools is expected to quantify contextual or nonspecific factors associated with healing while requiring no more than a total of 10–12 minutes of participants' time.

The HEAL CATs and Short Forms have the advantage of flexibility. Researchers and clinicians will be able to choose specific domains of interest for their study or clinical use rather than require patients to complete the entire set of instruments. For example, a study of yoga for pain management may include expectancy, patient-provider relationship, and spirituality CATs, but would not need to include perceptions of the health care environment, since yoga classes are not typically conducted in health care clinics. Thus, patient burden can be further minimized.

## 9. Conclusion

Although measurement challenges exist, a set of tools that can quantify nonspecific factors in healing outcomes will be an important step toward NCCAM's goal of improving research design and methods as outlined in the 3rd strategic plan. The HEAL instruments will have important implications for CAM clinical trials and also more broadly for chronic disease management. The tools will be a concise set of measures that are feasible for use across CAM, and presumably, conventional medicine trials. Precise measurement of nonspecific factors has the potential to change RCT methodology dramatically. The HEAL instruments can be used to determine the proportion of variability in treatment response that is due to patient attitudes, expectancies, and perceptions of patient-provider relationships and the overall healing environment. The ability to quantify patient attitudes and perceptions on the same metric, across health conditions and across treatment modalities, not only has the potential to dismantle “placebo” effects to some extent but also could be useful in comparative effectiveness studies of CAM. Also, because this project is synergistic with the PROMIS initiative, we expect that the measures will be readily available to administer electronically from diverse geographic sites. Additionally, because management of chronic diseases involves ongoing relationships with treatment providers as well as patients taking an active role in their health, the HEAL contextual factors tools could be important for determining which aspects of the healing context should be enhanced, and for whom.

## Figures and Tables

**Table 1:** Examples of item rewriting during item bank development phase of the HEAL project.

Domain	Original question	Rationales for rewriting	Revised item
Positive and negative attitudes	I often feel lonely because I have few close friends with whom to share my concerns.	Double-barreled item (several concepts within one question)	I feel lonely.

Treatment expectancy/credibility	I do not think the chiropractor can help me.	Negatively worded item, specific to one treatment provider/modality	I expect this treatment to help me.

Patient-provider encounter	I trust my therapist.	Specific to one provider	I trust my healthcare provider.

Locus of control/self-efficacy	In this life what happens to me is determined by fate.	Wordy	My life is influenced by fate.
